# KL Divergence-Based Fuzzy Cluster Ensemble for Image Segmentation

**DOI:** 10.3390/e20040273

**Published:** 2018-04-12

**Authors:** Huiqin Wei, Long Chen, Li Guo

**Affiliations:** Department of Computer and Information Science, Faculty of Science and Technology, University of Macau, Macau 999078, China

**Keywords:** fuzzy clustering, ensemble learning, KL divergence, spatial information, image segmentation

## Abstract

Ensemble clustering combines different basic partitions of a dataset into a more stable and robust one. Thus, cluster ensemble plays a significant role in applications like image segmentation. However, existing ensemble methods have a few demerits, including the lack of diversity of basic partitions and the low accuracy caused by data noise. In this paper, to get over these difficulties, we propose an efficient fuzzy cluster ensemble method based on Kullback–Leibler divergence or simply, the KL divergence. The data are first classified with distinct fuzzy clustering methods. Then, the soft clustering results are aggregated by a fuzzy KL divergence-based objective function. Moreover, for image segmentation problems, we utilize the local spatial information in the cluster ensemble algorithm to suppress the effect of noise. Experiment results reveal that the proposed methods outperform many other methods in synthetic and real image-segmentation problems.

## 1. Introduction

Image segmentation has become increasingly important in a wide variety of applications like biomedical image analysis [1,2,3,4] and intelligent robotics [5]. Unfortunately, due to the variations and noise of images, finding a good image partition is still a great challenge, especially when we want to stress the semantic meanings of different regions. There is a growing number of methods available for image-segmentation problems over recent years [1,2,3,4,5,6,7,8]. Fuzzy approaches show considerable advantages among these methods by carefully handling the ubiquitous uncertainty and unknown noise in images. In contrast to hard segmentation methods, the fuzzy ones could retain much more information from the original data [9,10,11].

The fuzzy c-means (FCM) clustering algorithm is the best known one in fuzzy segmentation methods [12]. FCM derives the segmentation by iteratively minimizing a cost function that is dependent on the distances of image pixels to cluster centers in the feature domain. However, the standard FCM does not consider any spatial information in the image context, and hence suffers from high sensitivity to noise. Many extensions of the standard FCM have been proposed to suppress the effects of noise in images [9,10,13,14]. For example, the spatial neighborhood information is incorporated into the membership function of FCM for clustering in [9,10]. With so many available algorithms, one may obtain very different clustering results for a given dataset. Without ground truth, it is difficult to select the most suitable method for a given problem. In addition, most of existing algorithms require the specification of some parameters to obtain a decent grouping of the data.

Instead of choosing a decent setting of parameters, a particular algorithm, a good clustering configuration, or a special similarity measure that best suits a given problem, ensemble clustering can integrate results from multiple weak partition algorithms into a single robust and stable solution. The inputs of ensemble clustering are a set of data partitions. Previously, ensemble approaches for clustering problems have been studied extensively [15,16,17,18,19,20,21,22,23]. These ensemble methods generate many partitions by using the same method with various parameter settings, distinct methods, different inputs, or divergent feature sets. The final merged partition is obtained by approaches like majority voting or evidence accumulation [20].

For the fuzzy c-means and its extensions, their outputs are soft partitions of data. A soft partition assigns a degree of association of each instance to every cluster. So instead of a label vector for all the instances, in soft partition we have a matrix of memberships in which each instance has a membership vector that represents its belongingness to all clusters. Many ensemble methods have been proposed for the soft partitions [16,17,21], in which the most straightforward approach is conducting the fuzzy c-means over the membership matrices of different soft partitions. However, FCM uses squared Euclidean distance to measure the similarity of a membership vector to a cluster center. This is inappropriate for the situation when one data’s memberships to all clusters usually sum to one [21]. A better solution is to regard the membership vector as some discrete probability function and use the statistical distance like KL divergence as the similarity measure [24].

In this paper, we first propose an efficient fuzzy cluster ensemble method based on KL divergence $\left(FCE_{\_KL}\right)$. This algorithm is similar to the fuzzy c-means, differing only in the fact that it uses the KL divergence to handle the memberships like discrete probabilities. Theoretically, we have developed an optimization algorithm for the proposed $FCE_{\_KL}$. For image-segmentation problems, because it is well known that the comparative performance of different clustering methods can vary significantly across datasets, we first utilize heterogeneous center-based soft clustering algorithms to categorize the pixels in the image. The soft clustering results obtained by different methods guarantee the diversity of partitions. Then, the fusion of soft partitions is provided by applying $FCE_{\_KL}$. Although $FCE_{\_KL}$ basically outperforms individual clustering methods, it still classifies noisy pixels in wrong segments sometimes. So, we further use the local spatial information in the calculation of membership values for $FCE_{\_KL}$ to enhance the accuracy of image segmentation and propose the spatial $FCE_{\_KL}$ ($FCE_{\_sKL}$). Experimental results on synthetic and real image segmentation demonstrate that the proposed methods perform better than some widely used fuzzy clustering-based approaches.

The remainder of this paper is organized as follows. In addition to several standard fuzzy clustering methods, Section 2 presents related ensemble clustering methodology that includes the ensemble clustering generator and consensus function. In Section 3, we propose the fuzzy cluster ensemble method based on KL divergence $\left(FCE_{\_KL}\right)$. For the image-segmentation problems, we utilize the local spatial information of the image to handle the membership values for the proposed clustering ensemble algorithm and propose $FCE_{\_sKL}$. In Section 4, the numerical experiments demonstrate the good performance of the proposed algorithm for image segmentation. Section 5 gives the discussion of our methods. At last, the conclusion and future work are given in Section 6.

## 2. Related Work

### 2.1. Fuzzy C-Means

Fuzzy c-means (FCM) [12] divides a set of *n* datapoints $x_{k}$ (k=1,2,…,n) into *c* clusters by minimizing the weighted summation of distances from the datapoints to the cluster centers:

(1) $$J=\sum_{k=1}^{n}\sum_{i=1}^{c}{\left(u_{ik}\right)}^{m}∥x_{k}-v_{i}{∥}^{2},$$

where ∥·∥ denotes the Euclidean distance, m>1 is the fuzzification coefficient, which usually takes the value of 2, $u_{ik}$ is the membership value of data $x_{k}$ to the *i*-th cluster center $v_{i}$ and $\sum_{i=1}^{c}u_{ik}=1$, $u_{ik}\in \left[0,1\right]$. The FCM algorithm iteratively updates $v_{i}$ and $u_{ik}$ as follows:

(2) $$v_{i}=\sum_{k=1}^{n}{\left(u_{ik}\right)}^{m}x_{k}/\sum_{k=1}^{n}{\left(u_{ik}\right)}^{m}$$

and

(3) $$u_{ik}=1/\sum_{j=1}^{c}{\left(\frac{∥x_{k}-v_{i}∥}{∥x_{k}-v_{j}∥}\right)}^{2/m-1},$$

until the stop criterion like maximum number of iterations is satisfied.

### 2.2. Local Spatial Fuzzy C-Means

To deal with noise and segment images better, one extension to FCM is incorporating the local spatial information into the standard FCM. In [25], the membership $u_{ij}$ is updated by the weighted average value of its neighbors’ membership values, which exploits the spatial information as the following:

(4) $${\hat{u}}_{ik}=\left(\sum_{\omega \in NB(x_{k})}\frac{1}{|NB|}u_{i\omega }+u_{ik}\right)/2,$$

where $NB(x_{k})$ denotes a local square window centered on pixel $x_{k}$ in the spatial domain, and ∣NB∣ is the size of the neighborhood.

On the one hand, clustering algorithms, such as FCM and its extensions, are effective for image segmentation. They could show good performance in some problems. On the other hand, cluster ensembles based on different clustering methods are more robust and stable. So, the proper combination of different fuzzy clustering algorithms could produce more reliable and accurate results.

### 2.3. Cluster Ensemble

Basically, there are two parts in the cluster ensembles. One is the ensemble clustering generator, and another is the consensus function. The first part concentrates on producing more diverse clustering results, while the second part concentrates on finding a good consensus function to improve the accuracy of the results. For the first part of cluster ensembles, Rathore et al. [17] proposed that multiple partitions can be obtained using the fuzzy c-means clustering algorithm on a randomly projected dataset. According to [18], Fred and Jain focused on running k-means several times by using different initializations to obtain various partitions. In [16], Zou et al. utilized different clustering algorithms to generate base partitions. For the second part of cluster ensembles, many methods have been proposed to fuse multiple partitions into a consensus one. The ensemble clustering method in [26] constructs the co-association matrix obtained by reliable data pairs in the same cluster among multiple partitions, and then applies the spectral clustering on the completed co-association matrix to obtain the best partition. In [27], the authors introduced an ensemble approach for categorical data by finding the best partition that minimizes an objective function. Similarly, ensemble clustering in [28] was cast into a problem that selects a consensus partition by maximizing the within-cluster similarity. Recently, Wu et al. proposed the utility function based on the fuzzified contingency matrix to measure the similarity multiple partitions. They established a framework for fuzzy consensus clustering by vertical and horizontal segmentation schemes to deal with big data clustering [23].

To solve the ensemble clustering problem, we can work on two basic operating units: the data objects and the basic clusters. Many effective algorithms have been proposed to handle the ensemble clustering problem at the object level [16,18,29] and cluster level [21,24]. In [24], each instance in a soft ensemble is represented by the concatenation of membership probability distributions. Then, a distance measure between two instances was defined using the Kullback–Leibler (KL) divergence. As the ensemble size becomes larger and the total number of clusters increases, the distance measure between clusters will be a computational burden. In contrast to cluster level, the data objects are used as basic operating units in this paper. We measure the similarly of a membership vector to a cluster center using the Kullback–Leibler (KL) divergence.

## 3. KL Divergence-Based Fuzzy Cluster Ensemble

In this section, we illustrate the problem of combining multiple clustering operations and propose an efficient fuzzy cluster ensemble method based on KL divergence. Then, when solving image-segmentation problems, we utilize the local spatial information of the image to handle the membership values for the proposed ensemble approach.

### 3.1. Formulation of the Fuzzy Cluster Ensemble Problem

In this paper, to build the ensemble, we first apply some heterogeneous center-based soft clustering algorithms to generate membership matrices as basic partitions ${\left\{U_{f}^{T}\right\}}_{f=1}^{r}$, where *r* is the number of soft clustering algorithms, and $U_{f}^{T}$ is the transposed membership matrix obtained by the *f*-th clustering method. In the membership matrix, each entry denotes the degree of data belonging to a cluster. Here, the number of basic partitions is the same as the one of clustering algorithms, and any clustering algorithm that generates membership matrices can be applied.

These partition matrices are then concatenated to $U^{\mathrm{con}}=\left[U_{1}^{T},\ldots ,U_{r}^{T}\right]\in R^{n\times s}$. Here, *n* is the number of the data and s is the number of memberships derived for data from different algorithms. If each partition has *c* clusters, we have s=cr for brevity. However, this is not a necessity, and we can have different number of clusters in different partitions. Let ${\hat{U}}^{\mathrm{con}}=\frac{1}{r}U^{\mathrm{con}}$ for the normalization of matrix $U^{\mathrm{con}}$ and we define ${\hat{u}}_{kj}^{\mathrm{con}}$ as the entry of ${\hat{U}}^{\mathrm{con}}$, where k=1,2,…,n;j=1,2,…,s. Let ${\hat{u}}_{k\cdot }^{\mathrm{con}}=\left({\hat{u}}_{k1}^{\mathrm{con}},{\hat{u}}_{k2}^{\mathrm{con}},\ldots ,{\hat{u}}_{ks}^{\mathrm{con}}\right)$, we have $∥{\hat{u}}_{k\cdot }^{\mathrm{con}}{∥}_{1}=1$, ∀k, which implies each row of ${\hat{U}}^{\mathrm{con}}$ as a probability vector. $\left\{{\hat{u}}_{kj}^{\mathrm{con}}\right\}$ is the input data of the $FCE_{\_KL}$ algorithm.

As an illustrated example, Table 1 demonstrates two soft basic partitions derived from two algorithms, and their concatenation $U^{\mathrm{con}}$ and normalization ${\hat{U}}^{\mathrm{con}}$. From this example, it is easy to know that we do not arrange lexicographically the different soft cluster solutions. In the proposed ensemble method, we simply concatenate and normalize all membership values of different fuzzy clustering methods and take them as the new representation of the data. The new representation sums to one and can be regarded as a discrete distribution. Therefore, the entropy-based KL divergence is a better measurement of a discrete distribution.

**Figure 1:**
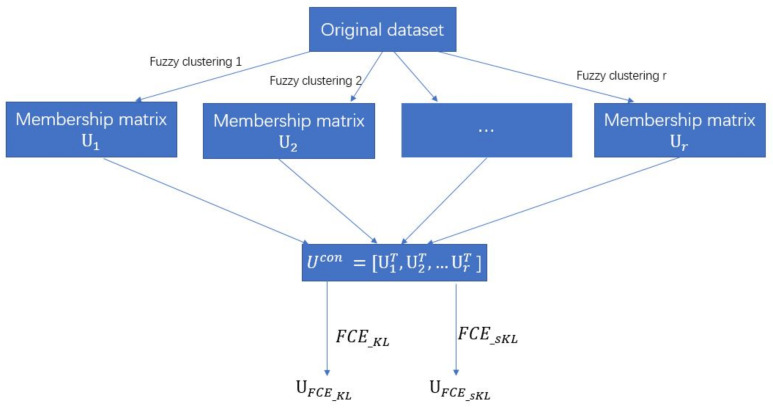
The framework of $FCE_{KL}$ and $FCE_{sKL}$.

We aggregate the soft clustering results by a fuzzy KL divergence-based objective function. For a new set of *n* probability vectors, $FCE_{\_KL}$ is applied to divide them into a desired number of clusters again. Specifically, $FCE_{\_KL}$ use KL divergence to measure the distance of a membership vector to a cluster center. Moreover, for the image-segmentation problems, we utilize the local spatial information for calculating the membership value of the proposed $FCE_{\_KL}$ and developed $FCE_{\_sKL}$. In the long run, we can get the ensemble clustering results by $FCE_{\_KL}$ and $FCE_{\_sKL}$. Figure 1 illustrates the framework of the two methods we proposed.

### 3.2. Fuzzy Cluster Ensemble Based on KL Divergence ($FCE_{\_KL}$)

The KL divergence is used to measure variation in the discrete probability distributions of attributes *P* and *Q*. It is defined by

(5) $$D_{KL}=\sum_{j}=P\left(j\right)\log\frac{P(j)}{Q(j)}.$$

Let $\left\{y_{kj}\right\}=\left\{{\hat{u}}_{kj}^{\mathrm{con}}\right\}$(k=1,2,…,n;j=1,2,…,s). The $FCE_{KL}$ divides a set of *n* probability vectors $y_{kj}$ with *s* dimension into *c* clusters by minimizing the following objective function,

(6) $$\begin{array}{cc} J= & \sum_{k=1}^{n}\sum_{i=1}^{c}u_{ik}^{m}D_{KL}\left(y_{kj}∥o_{ij}\right), \end{array}$$

subject to

(7) $$\sum_{i=1}^{c}u_{ik}=1,\;k=1,2,\ldots ,n,$$

(8) $$\sum_{j=1}^{s}o_{ij}=1,\;i=1,2,\ldots ,c,$$

where $u_{ik}$ presents the membership of the *k*-th probability vector in the *i*-th cluster $o_{i}\;\left(o_{i}=\left[o_{i1},\ldots ,o_{is}\right]\right)$, and $D_{KL}\left(y_{kj}∥o_{ij}\right)$ denotes $\sum_{j=1}^{s}y_{kj}\log\frac{y_{kj}}{o_{ij}}$. The vectors close to the centroid of their clusters based on KL divergence are assigned high membership values, and low membership values are assigned to datapoints far from the centroid to minimize the objective function.

Next, we take an iterative way for solving the modified membership and cluster centers here. Let the Lagrangian of formula (6) be

(9) $$\bar{J}=\sum_{k=1}^{n}\sum_{i=1}^{c}u_{ik}^{m}D_{KL}\left(y_{kj}∥o_{ij}\right)+\sum_{k=1}^{n}\alpha_{k}\left(\sum_{i=1}^{c}u_{ik}-1\right)+\sum_{i=1}^{c}\beta_{i}\left(\sum_{j=1}^{s}o_{ij}-1\right).$$

Letting the first derivatives of $\bar{J}$ with respect to *u* and *o* equal to zero yields the two necessary conditions for minimizing $\bar{J}$. Thus, we obtain

(10) $$\frac{\partial \bar{J}}{\partial o_{ij}}=\sum_{k=1}^{n}u_{ik}^{m}\left(-\frac{y_{kj}}{o_{ij}}\right)+\beta_{i}=0,$$

(11) $$\frac{\partial \bar{J}}{\partial u_{ik}}=mu_{ik}^{m-1}D_{KL}\left(y_{kj}∥o_{ij}\right)+\alpha_{k}=0,$$

respectively.

Using (10),

(12) $$o_{ij}=\frac{\sum_{k=1}^{n}u_{ik}^{m}y_{kj}}{\beta_{i}}.$$

By formula (8), we have

(13) $$1=\sum_{h=1}^{s}o_{ih}=\sum_{h=1}^{s}\frac{\sum_{k=1}^{n}u_{ik}^{m}y_{kh}}{\beta_{i}},$$

(14) $$\beta_{i}=\sum_{h=1}^{s}\sum_{k=1}^{n}u_{ik}^{m}y_{kh}.$$

Hence,

(15) $$o_{ij}=\frac{\sum_{k=1}^{n}u_{ik}^{m}y_{kj}}{\sum_{h=1}^{s}\sum_{k=1}^{n}u_{ik}^{m}y_{kh}}.$$

On the other hand, from (11),

(16) $$u_{ik}={\left(-\frac{\alpha_{k}}{m}\right)}^{\frac{1}{m-1}}\frac{1}{{\left(D_{KL}\left(y_{kj}∥o_{ij}\right)\right)}^{\frac{1}{m-1}}}.$$

Therefore, by formula (7), it holds that

(17) $$1=\sum_{\ell =1}^{c}u_{\ell k}=\sum_{\ell =1}^{c}{\left(-\frac{\alpha_{k}}{m}\right)}^{\frac{1}{m-1}}\frac{1}{{\left(D_{KL}\left(y_{kj}∥o_{\ell j}\right)\right)}^{\frac{1}{m-1}}},$$

(18) $${\left(-\frac{\alpha_{k}}{m}\right)}^{\frac{1}{m-1}}=\frac{1}{\sum_{\ell =1}^{c}\frac{1}{{\left(D_{KL}\left(y_{kj}∥o_{\ell j}\right)\right)}^{\frac{1}{m-1}}}},$$

(19) $$u_{ik}=\frac{1}{\sum_{\ell =1}^{c}{\left(\frac{D_{KL}\left(y_{kj}∥o_{ij}\right)}{D_{KL}\left(y_{kj}∥o_{\ell j}\right)}\right)}^{\frac{1}{m-1}}}.$$

Therefore, the $FCE_{\_KL}$ algorithm iteratively updates (15) and (19) until the stop criterion, like the convergence of the objective function or the satisfaction of maximum iteration number.

### 3.3. Spatial Information-Based $FCE_{\_KL}$

The disadvantage of $FCE_{\_KL}$ for image segmentation is the discarding of spatial information in images. Since neighboring pixels are highly correlated, to suppress the noise and acquire better segments, we include this property into the $FCE_{\_KL}$ algorithm. The membership of *k*-th pixel in the *i*-th cluster is summed over the weighted average value of the membership values of the user-specified neighbors as following [25]:

(20) $${\hat{u}}_{ik}=\left(\sum_{\omega \in NB(y_{k})}\frac{1}{|NB|}u_{i\omega }+u_{ik}\right)/2,$$

where $NB(y_{k})$ denotes a local square window centered on pixel $y_{k}$ in the spatial domain, and ∣NB∣ is the size of the neighborhood. The modified memberships can be used to get the cluster center by

(21) $${\hat{o}}_{ij}=\frac{\sum_{k=1}^{n}{\hat{u}}_{ik}^{m}y_{kj}}{\sum_{h=1}^{s}\sum_{k=1}^{n}{\hat{u}}_{ik}^{m}y_{kh}}.$$

Therefore, our spatial information-enhanced $FCE_{\_sKL}$ algorithm for image segmentation is presented in Algorithm 1. In the algorithm, the memberships and cluster centers are updated iteratively according to Equations (19)–(21).

**Algorithm 1:**
$FCE_{\_sKL}$
**Input:**   Normalized partitions ${\hat{U}}^{\mathrm{con}}$, values of the fuzzification coefficient *m*, maximum iteration number $t_{s}$ and a small enough error ϵ **Output:**  The consensus clustering;
1:Make initialization of cluster centers, ${\hat{o}}_{ij}$, i=1,2,…,c,j=1,2,…,s;2:t←1; 3:**repeat**4:Calculate the membership $u_{ik}$ by Equation (19);5:Utilize the spatial information in membership function of $u_{ik}$ and calculate new membership ${\hat{u}}_{ik}$ with Equation (20);6:Update cluster centers ${\hat{o}}_{ij}$ by Equation (21);7:Calculate the objective function $J^{\left(t\right)}$ by (6);8:t←t+1; 9:**until** $|J^{\left(t\right)}-J^{\left(t-1\right)}|<\epsilon$ or $t>t_{s}$


## 4. Experiment Results

In this section, we compare the newly proposed $FCE_{\_KL}$, $FCE_{\_sKL}$ with SFCM [25], SSCM [25], NLSFCM [16] and NLSSCM [16] on several synthetic and real images. For our clustering ensemble approaches, to generate basic partitions, we directly use SFCM, SSCM, NLSFCM and NLSSCM methods. Since *r* is the number of above-mentioned heterogeneous center-based clustering algorithms, here we have r=4. More specifically, the $FCE_{\_KL}$ and $FCE_{\_sKL}$ incorporate membership matrices derived by SFCM, SSCM, NLSFCM and NLSSCM to get their soft partitions. The fuzzification coefficient *m* is set to 2 for all the algorithms in our experiments. The size of square window NB of $FCE_{\_sKL}$ is set to 5×5. More settings and their justifications are provided in the following discussion section.

When the algorithms are tested on images with ground truth or reference partitions, the segmentation accuracy (SA) is calculated as

(22) $$Segmentation\;accuracy=\frac{number\;of\;correctly\;classfied\;pixels}{total\;number\;of\;pixels}.$$

The SA of algorithm *i* on class *j* is measured as

(23) $$SA_{j}^{i}=\frac{A_{j}^{i}\cap Aref_{j}}{A_{j}^{i}\cup Aref_{j}},$$

where $A_{j}^{i}$ denotes the set of pixels belonging to class *j* that are found by algorithm *i*, and $Aref_{j}$ denotes the set of pixels in class *j* which is in the reference segmented image. It should be noted that when we have a soft partition based on a fuzzy approach, we need a defuzzification method to assign each pixel to a segment. After we obtain the membership matrix $u_{ik}$, we calculate the $arg_{i}\left(max\left(u_{ik}\right)\right)$ to obtain the final results. In other words, we finally classify one pixel or data into the category where it takes the largest membership value.

### 4.1. Synthetic Images

**Figure 2:**
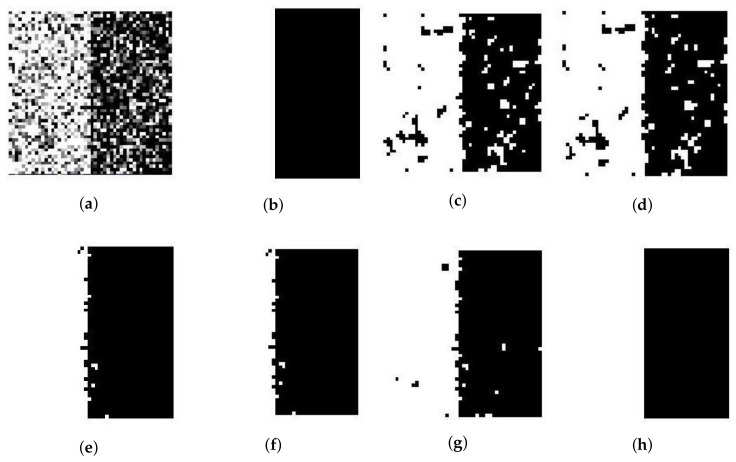
50% Gaussian-noised synthetic two-value image: (**a**) original; (**b**) ground-truth image and after segmentation into two regions with (**c**) SFCM; (**d**) SSCM; (**e**) NLSFCM; (**f**) NLSSCM; (**g**) $FCE_{\_KL}$; (**h**) $FCE_{\_sKL}$.

**Figure 3:**
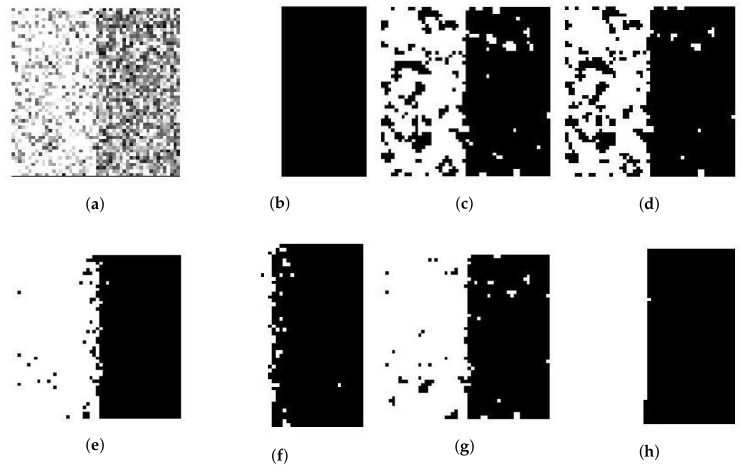
50% Rician-noised synthetic two-value image: (**a**) original; (**b**) ground-truth image and after segmentation into two regions with (**c**) SFCM; (**d**) SSCM; (**e**) NLSFCM; (**f**) NLSSCM; (**g**) $FCE_{\_KL}$; (**h**) $FCE_{\_sKL}$.

**Figure 4:**
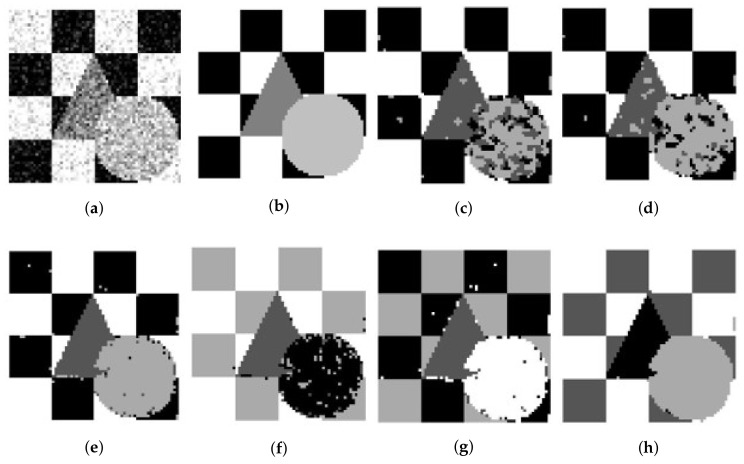
15% Gaussian-noised synthetic images of ‘Trin’: (**a**) original; (**b**) ground-truth image and after segmentation into four regions with (**c**) SFCM; (**d**) SSCM; (**e**) NLSFCM; (**f**) NLSSCM; (**g**) $FCE_{\_KL}$; (**h**) $FCE_{\_sKL}$.

**Figure 5:**
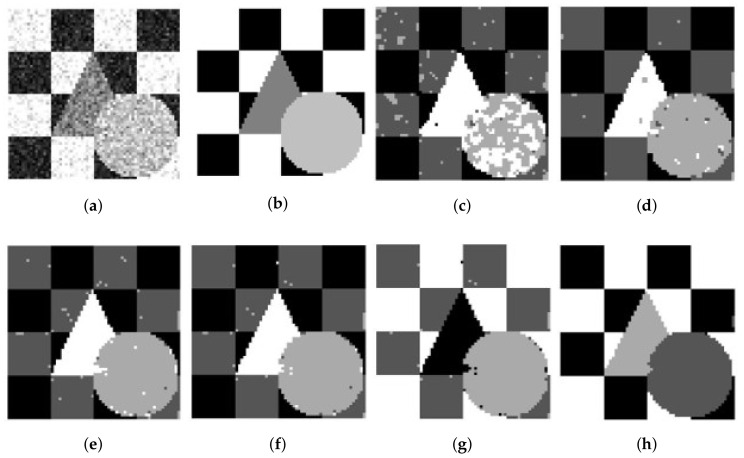
12% Rician-noised synthetic images of ‘Trin’: (**a**) original; (**b**) ground-truth image and after segmentation into four regions with (**c**) SFCM; (**d**) SSCM; (**e**) NLSFCM; (**f**) NLSSCM; (**g**) $FCE_{\_KL}$; (**h**) $FCE_{\_sKL}$.

We use a synthetic two-value image, the image ‘Trin’, and the synthetic magnetic resonance (MR) images as testing images first. The synthetic two-cluster image shown in Figure 2b, with values 1 and 0, is similar to the image used in [14]. Its size is set to 50×50 pixels. The synthetic image of ‘Trin’ contains four regions and its size is set to 64×64 pixels. Both synthetic images add Gaussian noise and Rician noise. Figure 2 and Figure 3a present synthetic two-value images with 50% Gaussian noise and 50% Rician noise. Figure 4 and Figure 5a show ‘Trin’ with 15% Gaussian noise and 12% Rician noise, respectively. For two sorts of noised images, Table 2 and Table 3 show the segmentation accuracies (SAs) of six methods. We obtain these results by running all methods on the same image (no matter the size or noise) for fair comparison.

For the synthetic two-value image, Table 2 shows that the performance of $FCE_{\_KL}$ and $FCE_{\_sKL}$ are as good as that of SFCM, SSCM, NLSFCM and NLSSCM, which means that all of them can easily remove the low-level noise from this image. When Gaussian noise reaches 30% and 50%, $FCE_{\_KL}$ can segment the image better than SFCM and SSCM but worse than NLSFCM and NLSSCM. $FCE_{\_sKL}$ can eliminate the noise better than the other five methods for the high-level noised images, and this demonstrates its robustness.

The fuzzy clustering methods, SFCM and SSCM, adjust the memberships with the local information of image. NLSFCM and NLSSCM update the membership values by the nonlocal information. Then, the four methods are all affected by the setting of neighborhood size and weight values when using the local or nonlocal information. In addition, NLSSCM and NLSFCM are affected much more by the initial values in the iteration than SFCM, SSCM and the proposed methods. Then, the simple setting of SFCM, SSCM and their robustness to the initialization may be the reason that some results of SFCM and SSCM in our experiments are the same as the ones in [25] coincidentally.

On the other hand, our experiments show different results of NLSFCM and NLSSCM when compared to [16], for we may choose a different size of nonlocal window, a different initialization, or a different computation of the nonlocal weights which are affected much by the similar measurement of patches in the nonlocal window. Some of our results of NLSSCM and NLSFCM are better than the ones in [16], and some are not. However, the difference is not great. So, the superiority of the proposed approach demonstrated in Table 2 is still trustworthy.

For the synthetic image “Trin”, Table 3 demonstrates that individual clustering methods (SFCM, SSCM, NLSFCM and NLSSCM) deteriorate dramatically with the increasing of noise rate. However, $FCE_{\_KL}$ and $FCE_{\_sKL}$ can stop this trend and improve the performance. $FCE_{\_KL}$ behaves better than individual clustering methods handling this image with Gaussian noise except 15% and 30%. $FCE_{\_KL}$ removes all Rician noise better than SFCM, SSCM, NLSFCM and NLSSCM. It is obvious that $FCE_{\_sKL}$ can handle this image with heavy Gaussian and Rician noise more easily than the other five methods. In order to visually compare the performance, the segmentation results of all of the six methods on the noised two-value image and the image “Trin” are shown in Figure 2, Figure 3, Figure 4 and Figure 5. All of these figures illustrate that $FCE_{\_sKL}$ behaves better than the other five methods.

**Figure 6:**
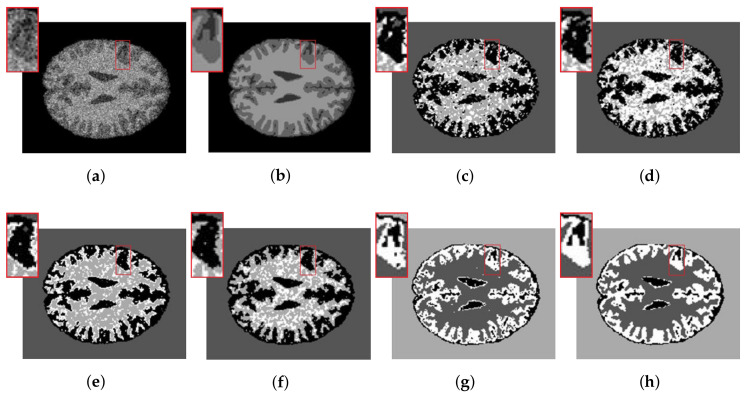
32% Rician-noised synthetic MR images: (**a**) original; (**b**) ground-truth image and after segmentation into four regions with (**c**) SFCM; (**d**) SSCM; (**e**) NLSFCM; (**f**) NLSSCM; (**g**) $FCE_{\_KL}$; (**h**) $FCE_{\_sKL}$.

The synthetic MR images of the human brain and their reference segmentations are provided by [30]. They are T1-weighted MR phantom with slice thickness of 1 mm, having various levels of Rician noise, without intensity inhomogeneity. The Rician noise rates range from 25% to 50% added to the synthetic MR images of the human brain. Since ground truth of the synthetic MR image is available, Table 4 shows the SAs of the six methods. Both $FCE_{\_KL}$ and $FCE_{\_KL}$ segment these images better than SFCM, SSCM, NLSFCM and NLSSCM. Furthermore, according to the SAs, the superiority of $FCE_{\_sKL}$ can be verified easily. Figure 6a presents the synthetic synthetic MR images with 32% Rician noise. We add the zoom of the image portion highlighted with a red rectangle. Segmentation results in enlarged red rectangles reveal that $FCE_{\_KL}$ and $FCE_{\_sKL}$ can remove noise better and obtain smoother regions than the other four methods. $FCE_{\_sKL}$ behaves better than $FCE_{\_KL}$ because the latter still produces several misclassified pixels.

### 4.2. Real Images

**Figure 7:**
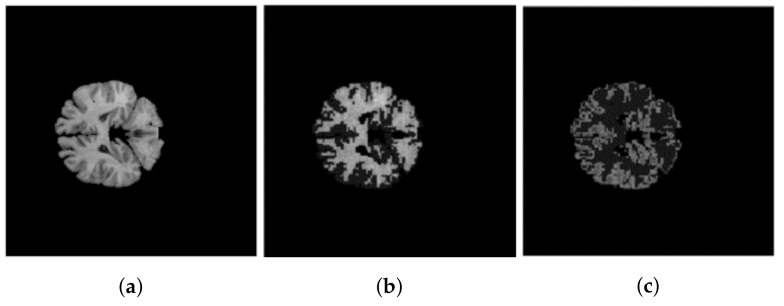
(**a**) Original IBSR image; (**b**) WM; (**c**) GM.

The first real image is the MR brain image obtained from the Internet Brain Segmentation Repository (IBSR) database [31]. This image should be partitioned into three regions corresponding to cerebrospinal fluid (CSF), white matter (WM) and gray matter (GM). As mentioned in [32], one general method of MR brain image segmentation involves two parts: the classification and the identification of all of the voxels belonging to a specific structure. Since the CSF in the center of the brain is a continuous volume, it can be well segmented by some contour-mapping algorithm. Therefore, this paper focuses on the segmenting of WM and GM. Figure 7 illustrates one sample without noise. According to the ground truth of the IBSR image, the SAs of different methods on the images having 12%, 15% and 18% Rician noise are shown in the Table 5, Table 6 and Table 7, in which $SA_{1}$ stands for SA for the cluster of WM, and $SA_{2}$ is for the cluster of GM. The Table 5, Table 6 and Table 7 demonstrate excellent performance of $FCE_{\_sKL}$ in terms of various levels of noise.

**Figure 8:**
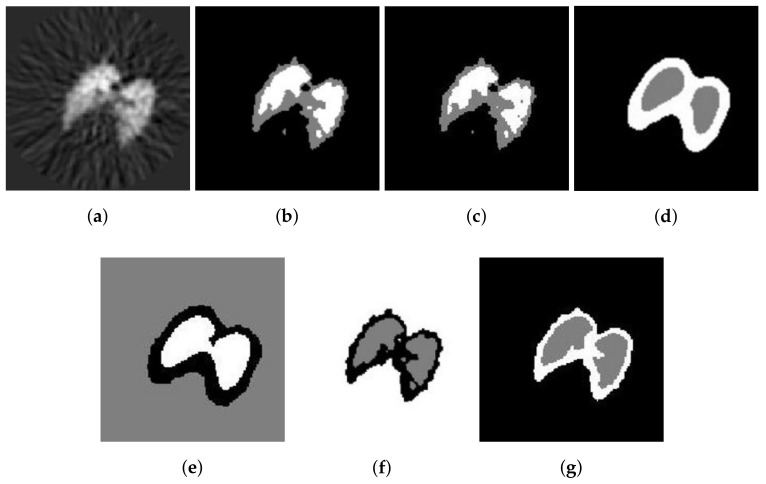
Segmentation result of different methods on a PET image of the lung of dog: (**a**) original and after segmentation into three regions with (**b**) SFCM; (**c**) SSCM; (**d**) NLSFCM; (**e**) NLSSCM; (**f**) $FCE_{\_KL}$; (**g**) $FCE_{\_sKL}$.

The second real image is the positron emission tomography (PET) lung image of a dog. It is demonstrated in Figure 8a, with 128×128 pixels. The reference segmentations of these images are not available. However, the segmentation results with six methods are illustrated in Figure 8b–g. Visually, the results obtained by SFCM and SSCM contain many flakes, while the results using NLSFCM and NLSSCM are more robust, such that some details of the lung are ignored. The Figure 8f,g reveals that $FCE_{\_KL}$ and $FCE_{\_sKL}$ outperform the other four methods. $FCE_{\_sKL}$ behaves better than $FCE_{\_KL}$, for $FCE_{\_KL}$ still produces several misclassified pixels.

**Figure 9:**
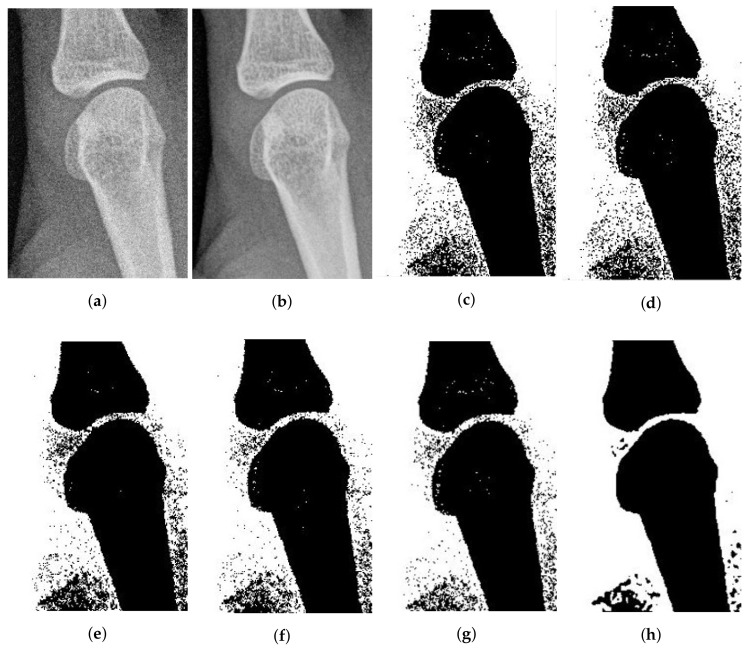
10% Gaussian-noised bone images: (**a**) original; (**b**) noise-free image and after segmentation into two regions with (**c**) SFCM; (**d**) SSCM; (**e**) NLSFCM; (**f**) NLSSCM; (**g**) $FCE_{\_KL}$; (**h**) $FCE_{\_sKL}$.

Another medical image is shown in Figure 9b, which is a 540×362 image of a healthy bone. We add 10% Gaussian noise to this image. From Figure 9, it is obvious that the performance of $FCE_{\_sKL}$ is better than the other five methods. $FCE_{\_sKL}$ can remove noise in the image and obtain more homogeneous regions.

**Figure 10:**
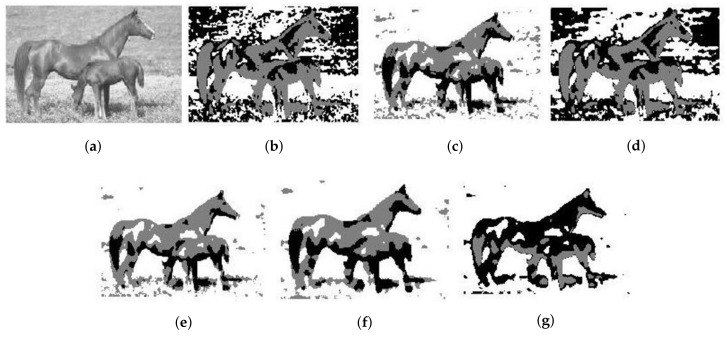
Segmentation result of different methods on an image of a horse: (**a**) original and after segmentation into three regions with (**b**) SFCM; (**c**) SSCM; (**d**) NLSFCM; (**e**) NLSSCM; (**f**) $FCE_{\_KL}$; (**g**) $FCE_{\_sKL}$.

The last real image is demonstrated in Figure 10a, which is a 160×240 image of horses. From Figure 10, SFCM and NLSFCM contain lots of misclassified pixels. In contrast, the segmentation results obtained by $FCE_{\_KL}$ and $FCE_{\_sKL}$ have less flakes in the lower-left corner than the other four methods. $FCE_{\_KL}$ and $FCE_{\_sKL}$ can well segment the horses from the background.

## 5. Discussion

In the previous section, compared with different clustering methods like SFCM, SSCM, NLSFCM and NLSSCM, both $FCE_{\_KL}$ and $FCE_{\_sKL}$ have shown superior performance in segmentation of low-level noised synthetic and real images. The possible explanation is that for image-segmentation problems, the results obtained by SFCM and SSCM contain many flakes, while the results using NLSFCM and NLSSCM are much more robust such that some details of the images are ignored easily. The ensemble methods like $FCE_{\_KL}$ and $FCE_{sKL}$ can avoid the weakness of a single clustering method. Experiment results also demonstrate that $FCE_{\_sKL}$ can eliminate the high-level noise better than all the other five methods in synthetic and real image segmentation. This is the result of $FCE_{\_sKL}$’s inclusion of spatial information for the purpose of noise suppression.

For the parameters of different clustering algorithms used by the ensemble, various settings can be chosen. However, in this paper we applied some ad-hoc selections. For example, the fuzzy coefficient *m* has huge influence on fuzzy clustering, notably in FCM. In contrast to k-means clustering, in fuzzy c-means, the datapoint is not directly assigned to any cluster, but its fuzzy membership values of all clusters are given as the final result. When *m* –> 1, FCM is similar to the k-means algorithm. The larger *m*, the more clusters share their objects, and vice versa. The fuzzification coefficient *m* was set to 2 in many studies. We also set m=2 in this paper. This decision should not discourage the future study on applying different fuzzy coefficients in fuzzy cluster ensembles, and more discussion on the determining of parameters for fuzzy c-means cluster analysis can be found in [33,34]. Similarly, we do not carefully select the parameters used in noise suppression of clustering algorithms. For example, the size of the local square window NB of $FCE_{\_sKL}$ is set to 5×5 in our experiments. If we choose a larger size of square window NB to include more local information and ignore the computing cost, the performance of $FCE_{\_sKL}$ may be better.

In unsupervised learning, the goodness-of-fit measure related to the number of cluster centers can be obtained by many methods [35,36]. Because it is not the focus of our research, we set it as the prior knowledge in this paper. For image-segmentation problems, the number of segmentation regions is usually expected to be known or given by the image directly in the experiments [14]. Moreover, in practical applications, it is not mandatory to get the same number of clusters across all different clustering methods in the ensemble, especially in the process of obtaining the basic partitions by different clustering methods for our proposed method. That is to say, different numbers of cluster centers can be used by these different clustering methods to get the basic partitions. In Figure 1, the obtained membership matrices, $U_{1},U_{2},\cdot \cdot \cdot U_{r}$, can be of different sizes in rows. However, in the experiments, we use the ground truth and the measurement SA to do fair comparisons. Because the number of clusters has great influence on the values of SA, the number of clusters must be set the same across all different clustering methods when dealing with the specified picture. Furthermore, we want to show the proposed ensemble methods outperform the other clustering methods in the case of the exact same parameter settings. So, the same number of clusters are used in all methods of the experiments.

## 6. Conclusions

In this paper, our main contribution is to propose an efficient fuzzy cluster ensemble method based on KL divergence, $\left(FCE_{\_KL}\right)$, considering that soft partitions are more suitable to be measured by KL divergence. In order to obtain ensemble partitions as diverse as possible, the data are classified using distinct clustering methods instead of a single method with different parameters. Theoretically, we have developed an optimization algorithm for the proposed $FCE_{\_KL}$. For image-segmentation problems, we further use spatial information in our method and propose $FCE_{\_sKL}$. According to experimental results, the proposed methods perform better than many existing clustering methods in synthetic and real image-segmentation problems.

In future work, we would explore more robust distance calculations instead of KL divergence in ensemble clustering. Currently, we handle each base clustering equally, which could overlook the different reliabilities of base clusterings. So, we will also explore certain methods to calculate weights of base clusterings for further consideration. 

## Figures and Tables

**Table 1:** Concatenation $U^{\mathrm{con}}$ and normalization ${\hat{U}}^{\mathrm{con}}$.

	$U_{1}$				$U_{2}$				$U^{\mathrm{con}}$							${\hat{U}}^{\mathrm{con}}$					
$x_{1}$	0.7	0.2	0.1		0.1	0.7	0.2		0.7	0.2	0.1	0.1	0.7	0.2		0.35	0.10	0.05	0.05	0.35	0.10
$x_{2}$	0.9	0.1	0.0		0.0	0.8	0.2		0.9	0.1	0.0	0.0	0.8	0.2		0.45	0.05	0.00	0.00	0.40	0.10
$x_{3}$	0.2	0.6	0.2		0.1	0.1	0.8		0.2	0.6	0.2	0.1	0.1	0.8		0.10	0.30	0.10	0.05	0.05	0.40
$x_{4}$	0.1	0.9	0.0		0.2	0.1	0.7		0.1	0.9	0.0	0.2	0.1	0.7		0.05	0.45	0.00	0.10	0.05	0.35
$x_{5}$	0.1	0.2	0.7		0.6	0.2	0.2		0.1	0.2	0.7	0.6	0.2	0.2		0.05	0.10	0.35	0.30	0.10	0.10

**Table 2:** Segmentation accuracies of different methods on noised two-value images.

SA	SFCM	SSCM	NLSFCM	NLSSCM	${\mathrm{FCE}}_{\_\mathrm{KL}}$	${\mathrm{FCE}}_{\_\mathrm{sKL}}$
1% Gaussian	1.0000	1.0000	1.0000	1.0000	1.0000	1.0000
3% Gaussian	1.0000	1.0000	1.0000	1.0000	1.0000	1.0000
5% Gaussian	1.0000	1.0000	1.0000	1.0000	1.0000	1.0000
10% Gaussian	0.9992	0.9992	0.9996	0.9996	0.9996	1.0000
15% Gaussian	0.9984	0.9980	0.9992	0.9992	0.9992	1.0000
20% Gaussian	0.9928	0.9932	0.9988	0.9988	0.9988	1.0000
30% Gaussian	0.9732	0.9760	0.9976	0.9976	0.9956	1.0000
50% Gaussian	0.9100	0.9148	0.9916	0.9916	0.9808	1.0000
1% Rician	1.0000	1.0000	1.0000	1.0000	1.0000	1.0000
3% Rician	1.0000	1.0000	1.0000	1.0000	1.0000	1.0000
5% Rician	1.0000	1.0000	1.0000	1.0000	1.0000	1.0000
10% Rician	1.0000	1.0000	1.0000	1.0000	1.0000	1.0000
15% Rician	1.0000	1.0000	1.0000	1.0000	1.0000	1.0000
20% Rician	1.0000	1.0000	1.0000	1.0000	1.0000	1.0000
30% Rician	0.9956	0.9960	0.9988	0.9988	0.9988	0.9996
50% Rician	0.8640	0.8664	0.9744	0.9828	0.9678	0.9968

**Table 3:** Segmentation accuracies of different methods on noised images of ‘Trin’.

SA	SFCM	SSCM	NLSFCM	NLSSCM	${\mathrm{FCE}}_{\_\mathrm{KL}}$	${\mathrm{FCE}}_{\_\mathrm{sKL}}$
10% Gaussian	0.9817	0.9878	0.9946	0.9951	0.9966	0.9968
12% Gaussian	0.9563	0.9712	0.9893	0.9817	0.9922	0.9951
15% Gaussian	0.8979	0.9348	0.9800	0.9670	0.9797	0.9907
18% Gaussian	0.8420	0.8896	0.9624	0.9138	0.9631	0.9836
20% Gaussian	0.8218	0.8457	0.9290	0.8937	0.9468	0.9785
25% Gaussian	0.7729	0.7773	0.7847	0.7813	0.9026	0.9558
30% Gaussian	0.6860	0.7446	0.7341	0.7539	0.7239	0.8569
10% Rician	0.9839	0.9917	0.9919	0.9934	0.9954	0.9973
12% Rician	0.8782	0.9768	0.9817	0.9888	0.9897	0.9951
15% Rician	0.7578	0.7136	0.9209	0.9670	0.9758	0.9905
18% Rician	0.7114	0.7112	0.7886	0.9526	0.9570	0.9832
20% Rician	0.6799	0.7085	0.7598	0.8413	0.9377	0.9729
25% Rician	0.6650	0.6775	0.7681	0.7531	0.8472	0.9263
30% Rician	0.6287	0.6406	0.6914	0.6818	0.7832	0.8472

**Table 4:** Segmentation accuracies of different methods on noised synthetic MR images.

SA	SFCM	SSCM	NLSFCM	NLSSCM	${\mathrm{FCE}}_{\_\mathrm{KL}}$	${\mathrm{FCE}}_{\_\mathrm{sKL}}$
25% Rician	0.8523	0.8125	0.8438	0.9543	0.9692	0.9700
27% Rician	0.8473	0.8117	0.8411	0.8504	0.9669	0.9670
30% Rician	0.8405	0.8070	0.8391	0.8411	0.9605	0.9632
32% Rician	0.8373	0.8026	0.8393	0.8339	0.9559	0.9608
35% Rician	0.8321	0.7966	0.8390	0.8317	0.9485	0.9566
40% Rician	0.8160	0.7903	0.8395	0.8249	0.9361	0.9493
50% Rician	0.7804	0.7784	0.8610	0.7911	0.9074	0.9298

**Table 5:** Segmentation accuracies of different methods on noised images of ‘IBSR12%’.

12% Rician Noise	SFCM	SSCM	NLSFCM	NLSSCM	${\mathrm{FCE}}_{\_\mathrm{KL}}$	${\mathrm{FCE}}_{\_\mathrm{sKL}}$
SA	0.7293	0.7400	0.7181	0.7163	0.7247	0.7526
$SA_{1}$	0.7463	0.7530	0.7297	0.7247	0.7381	0.7663
$SA_{2}$	0.7099	0.7256	0.7055	0.7073	0.7098	0.7372

**Table 6:** Segmentation accuracies of different methods on noised images of ‘IBSR15%’.

15% Rician Noise	SFCM	SSCM	NLSFCM	NLSSCM	${\mathrm{FCE}}_{\_\mathrm{KL}}$	${\mathrm{FCE}}_{\_\mathrm{sKL}}$
SA	0.7079	0.7237	0.7023	0.7093	0.7098	0.7442
$SA_{1}$	0.7281	0.7431	0.7248	0.7421	0.7369	0.7679
$SA_{2}$	0.6844	0.7012	0.6758	0.6670	0.6763	0.7150

**Table 7:** Segmentation accuracies of different methods on noised images of ‘IBSR18%’.

18% Rician Noise	SFCM	SSCM	NLSFCM	NLSSCM	${\mathrm{FCE}}_{\_\mathrm{KL}}$	${\mathrm{FCE}}_{\_\mathrm{sKL}}$
SA	0.6744	0.7014	0.6879	0.7028	0.6953	0.7307
$SA_{1}$	0.7031	0.7268	0.7205	0.7584	0.7338	0.7653
$SA_{2}$	0.6395	0.6708	0.6467	0.6139	0.6438	0.6841

## Abbreviations

The following abbreviations are used in this manuscript:

$FCE_{\_KL}$	Fuzzy cluster ensemble method based on KL divergence
$FCE_{\_sKL}$	Spatial $FCE_{\_KL}$
